# Supplementary material for: Recognizing hand use and hand role at home after stroke from egocentric video
Source: PLOS Digit Health. 2023 Oct 11;2(10):e0000361. doi: 10.1371/journal.pdig.0000361 (PMC10566743; doi:10.1371/journal.pdig.0000361)
Supplement: S1 Appendix — (PDF) [file pdig.0000361.s001.pdf]

S1 Appendix. A list of daily tasks carried out in the home simulation lab.

Dinning Room

1. Pick up a mug with liquid weight 350-400g.
2. Pick up a single sheet of standard paper from the round table.
3. Pick up a pencil from the round table.
4. Write a word with the pencil on the paper.
5. Pick up a book on the table, flip a few pages, and close it.
6. Pick up a credit card.
7. Swap the credit card on the mock machine.
8. Pick up a mobile phone.
9. Dial “123” on the mobile phone.
10. Use a key to open a lock.
11. Pick up a coin from the table and put it into a piggy bank.
12. Pick up a dice and toss it.
13. Pick up a nut from the table and screw it into the matching screw.
14. Open and close a Ziploc bag filled with 5 Golf balls on the round table.
15. Open web browser on a tablet, and type “google.ca".
16. Open and pick up a full pop can (355 ml) at the round table.
17. Pour 0.5 L of water from a bottle to the coffee cup.
18. Pretend to drink from the coffee cup.
19. Pick up and eat potato chips from a container.

20. Cut banana and eat with fork (banana already peel).

21. Clean hands with tissue.

22. Put your hand on the table and look around for 20 seconds.

23. Put your hand on side and look around for 20 seconds.

24. Right hand quick waves in the dinning room for 20 seconds.

25. Left hand quick waves in the dinning room for 20 seconds.

Kitchen

26. Open a disposable water bottle and pour some water into a disposable cup.

27. Grab a straw and place it in the cup, then drink from the cup.

28. Unscrew the lid of a Jar on the kitchen counter.

29. Pick up the sponge at the sink.

30. Open and close a container.

31. Open the fridge.

32. Put hands by the sink and look around for 20 seconds.

33. Put hands on the side, stand by the sink area and look around for 20 seconds.

34. Put hands on the counter area and look around for 20 seconds.

35. Put hands on the side, stand by the counter and look around for 20 seconds.

36. Right hand quick waves in the kitchen for 20 seconds.

37. Left hand quick waves in the kitchen for 20 seconds.

Washroom

38. Wash hands with hand soap in the washroom.

39. Pick up the toothbrush and pretend to clean teeth in the washroom.

40. Open and close the pill organizer.

41. Replace the empty tissue roll in the washroom.

42. Put hands on the washroom sink and look around for 20 seconds.

43. Put hands on the side, stand by the washroom sink, and look around for 20 seconds.

44. Right hand quick waves in the washroom for 20 seconds.

45. Left hand quick waves in the washroom for 20 seconds.

Living Room

46. Open a folded newspaper, pretend to read and fold back the newspaper.

47. Turn on and off the TV using the remote.

48. Type “google.ca” using a keyboard.

49. Put hands on laps while sitting on the sofa and look around the 20 seconds.

50. Put hands on laps and cover the hands with a pillow while sitting on the sofa and look around the 20 seconds.

51. Right hand quick waves in the living room for 20 seconds.

52. Left hand quick waves in the living room for 20 seconds.

Bedroom

53. Open the door in the bedroom.

54. Pick up the T-shirt from the bed and hang it up in the bar.

55. Fold a towel and place it in the drawer.

56. Sit on the bed with hands on laps and look around for 20 seconds.

57. Sit on the bed with hands on laps and cover the hands with a pillow, and look around for 20 seconds.

58. Right hand quick waves in the bedroom for 20 seconds.
59. Left hand quick waves in the bedroom for 20 seconds.

Hallway

60. Open the door on the hallway.
61. Place oranges (tennis balls) on the bench into a plastic bag.
62. Put one hand behind the back and the other hand on the bench, look around the bench area for 20 seconds.
63. Put both hands on the bench, look around the bench area for 20 seconds.
64. Right hand quick waves for 20 seconds.
65. Left hand quick waves for 20 seconds.
